# Exploring the anti-atherosclerosis mechanism of ginsenoside Rb1 by integrating network pharmacology and experimental verification

**DOI:** 10.18632/aging.205680

**Published:** 2024-03-27

**Authors:** Lianjie Hou, Zhiming Zou, Yu Wang, Hui Pi, Zeyue Yuan, Qin He, Yongfang Kuang, Guojun Zhao

**Affiliations:** 1 Affiliated Qingyuan Hospital, Guangzhou Medical University (Qingyuan People’s Hospital), Qingyuan 511518, Guangdong, China; 2 The Second Clinical Medical College, Guangzhou University of Chinese Medicine, Integrated Hospital of Traditional Chinese Medicine, Southern Medical University, Guangzhou 510120, Guangdong, China; 3 Dali University, Dali 671003, Yunnan, China

**Keywords:** ginsenoside Rb1, atherosclerosis, network pharmacology, CCND1

## Abstract

Ginsenoside Rb1 is the major active constituent of ginseng, which is widely used in traditional Chinese medicine for the atherosclerosis treatment by anti-inflammatory, anti-oxidant and reducing lipid accumulation. We explored cellular target and molecular mechanisms of ginsenoside Rb1 based on network pharmacology and *in vitro* experimental validation. In this study, we predicted 17 potential therapeutic targets for ginsenoside Rb1 with atherosclerosis from public databases. We then used protein-protein interaction network to screen the hub targets. Gene Ontology enrichment and Kyoto Encyclopedia of Genes and Genomes pathway enrichment showed that the effects of ginsenoside Rb1 were meditated through multiple targets and pathways. Next, molecular docking results revealed that in the 10 core targets, CCND1 has the highest binding energy with ginsenoside Rb1. Vascular cell proliferation plays a critical role in atherosclerosis development. However, the effect and direct target of ginsenoside Rb1 in regulating vascular cell proliferation in atherosclerosis remains unclear. Edu straining results indicated that ginsenoside Rb1 inhibited the cell proliferation of endothelial cells, macrophages, and vascular smooth muscle cells. The protein immunoprecipitation (IP) analysis showed that ginsenoside Rb1 inhibited the vascular cell proliferation by suppressing the interaction of CCDN1 and CDK4. These findings systematically reveal that the anti-atherosclerosis mechanism of ginsenoside Rb1 by integrating network pharmacology and experimental validation, which provide evidence to treat atherosclerosis by using ginsenoside Rb1 and targeting CCND1.

## INTRODUCTION

Atherosclerosis is the major cause of cardiovascular diseases (CVD), which results in a high mortality rate in the world [1]. Although medication and lifestyle management have made great progress, prevalence increases with age. Therefore, it is urgent to develop reliable therapy to alleviate or cure atherosclerosis and reduce the society burden. Panax ginseng has been used as a valuable herb over 2000 years in Eastern Asia [2]. As early as the 18th century, the Western world recognized the efficacy of ginseng, and ginseng has become the most popular herb in the world [3]. Ginsenosides are the major active substances in ginseng and widely used in traditional Chinese medicine to treat diverse CVD [4, 5]. Currently, more than 30 ginsenosides have been identified in ginseng and ginsenoside Rb1 is the most abundant ginsenoside, which constitutes about 0.5% of ginseng extracts [6]. The molecular formula of ginsenoside Rb1 is C_54_H_92_O_23_ with a tetracyclic triterpenoid structure, and the molecular weight is 1109.26 [7]. Modern pharmacological studies indicate that ginsenoside Rb1 enhanced the plaque stability of atherosclerotic mice through promoting M2 macrophage polarization and increasing the IL-4 and IL-13 production [8]. Ginsenoside Rb1 also enhanced atherosclerotic plaque stability by inducing AMPK-meditated macrophage autophagy [9]. Ginsenoside Rb1 enhanced plaque stability and attenuated plaque growth through regulating miR-33-meditated adventitial vasa vasorum proliferation and inflammation in ApoE^-/-^ mice [10]. However, neither miR-33 nor AMPK is the direct target of ginsenoside Rb1 for anti-atherosclerosis.

**Figure 1:**
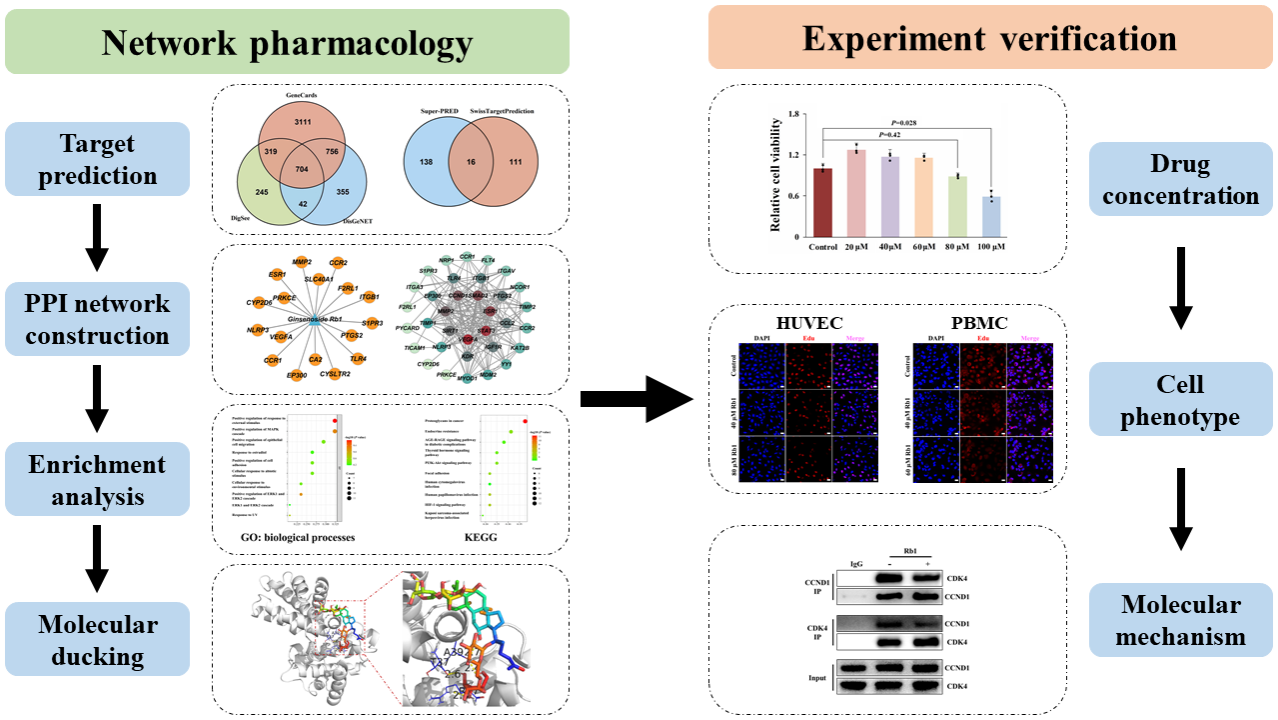
Flow chart of the present study.

Recently, network pharmacology has provided a new strategy to identify the regulating mechanisms by which drugs treat disease through bioinformatics, computer science, multidirectional pharmaceutical biology, and integrating systems biology [11, 12]. Previous ginsenoside Rb1 studies in atherosclerosis were focused on its function and regulatory signaling pathway, there is a lack of network pharmacology analysis and experimental verification. Hence, we have conducted a comprehensive network pharmacology analysis and *in vitro* studies to further clarify the therapeutic targets and potential mechanisms of ginsenoside Rb1 in treating atherosclerosis. In Figure 1, we showed the flow chart of this study.

## RESULTS

### Screen potential targets of ginsenoside Rb1 in treating atherosclerosis

**Figure 2:**
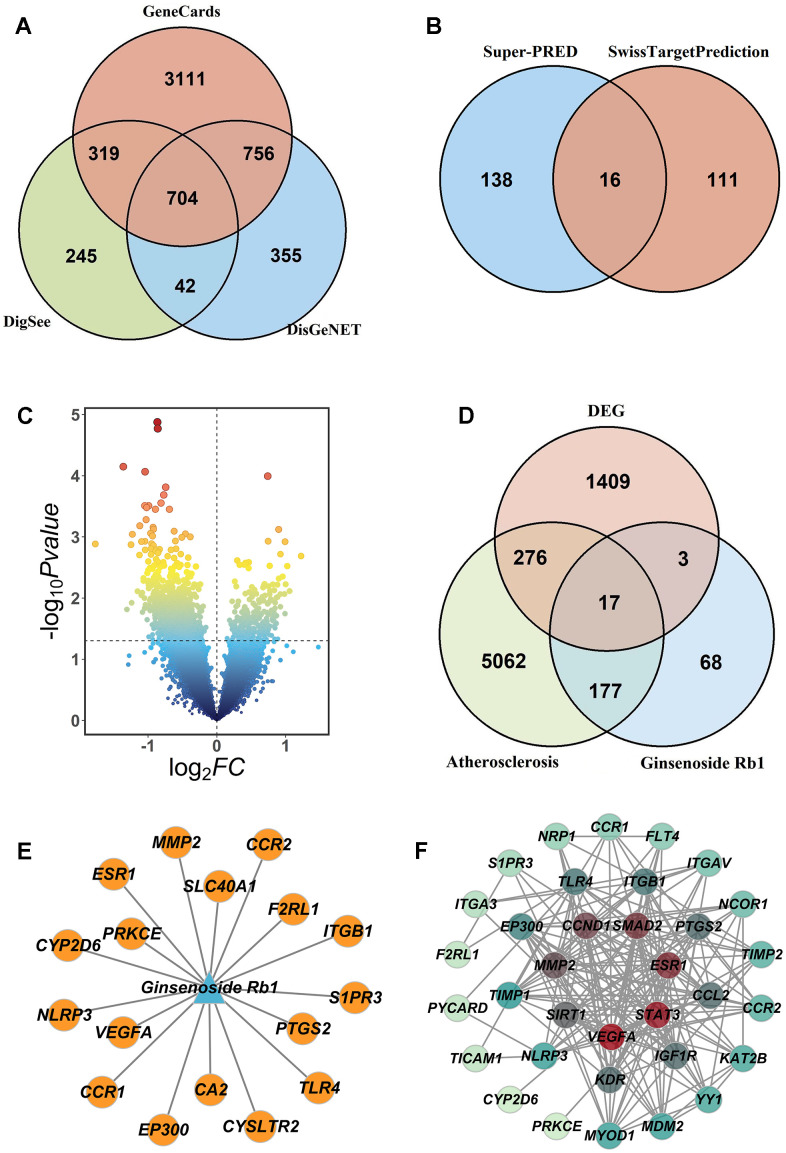
**Screen potential targets of ginsenoside Rb1 in the treatment of atherosclerosis.** (**A**) The atherosclerosis-related targets were shown by Venn diagram. (**B**) The putative targets of ginsenoside Rb1 were shown by Venn diagram. (**C**) The differentially expressed genes in atherosclerosis were shown by Volcano plot. (**D**) The comment targets of ginsenoside Rb1 and atherosclerosis were shown by Venn diagram. (**E**) Comment targets of ginsenoside Rb1 and atherosclerosis. (**F**) PPI network of ginsenoside Rb1 was used to show the hub genes.

We obtained 4890 targets in Genecards database, 1310 targets in DigSee database, 1857 targets in DisGeNET database, and got 5532 atherosclerosis-related targets in the three databases (Figure 2A). Through deleting duplicate entries in the Super-PRED database and Swiss Target Prediction database, we identified 265 putative targets of ginsenoside Rb1 (Figure 2B). The GEO database (GSE202625, atherosclerosis patients) showed 1705 differential genes between the disease group and the control group (Figure 2C). Then, 5532 atherosclerosis-related targets, 265 drug targets and 1705 differential genes in atherosclerosis were intersected to obtain 17 ginsenoside Rb1 targets during atherosclerosis treatment (Figure 2D).

**Figure 3:**
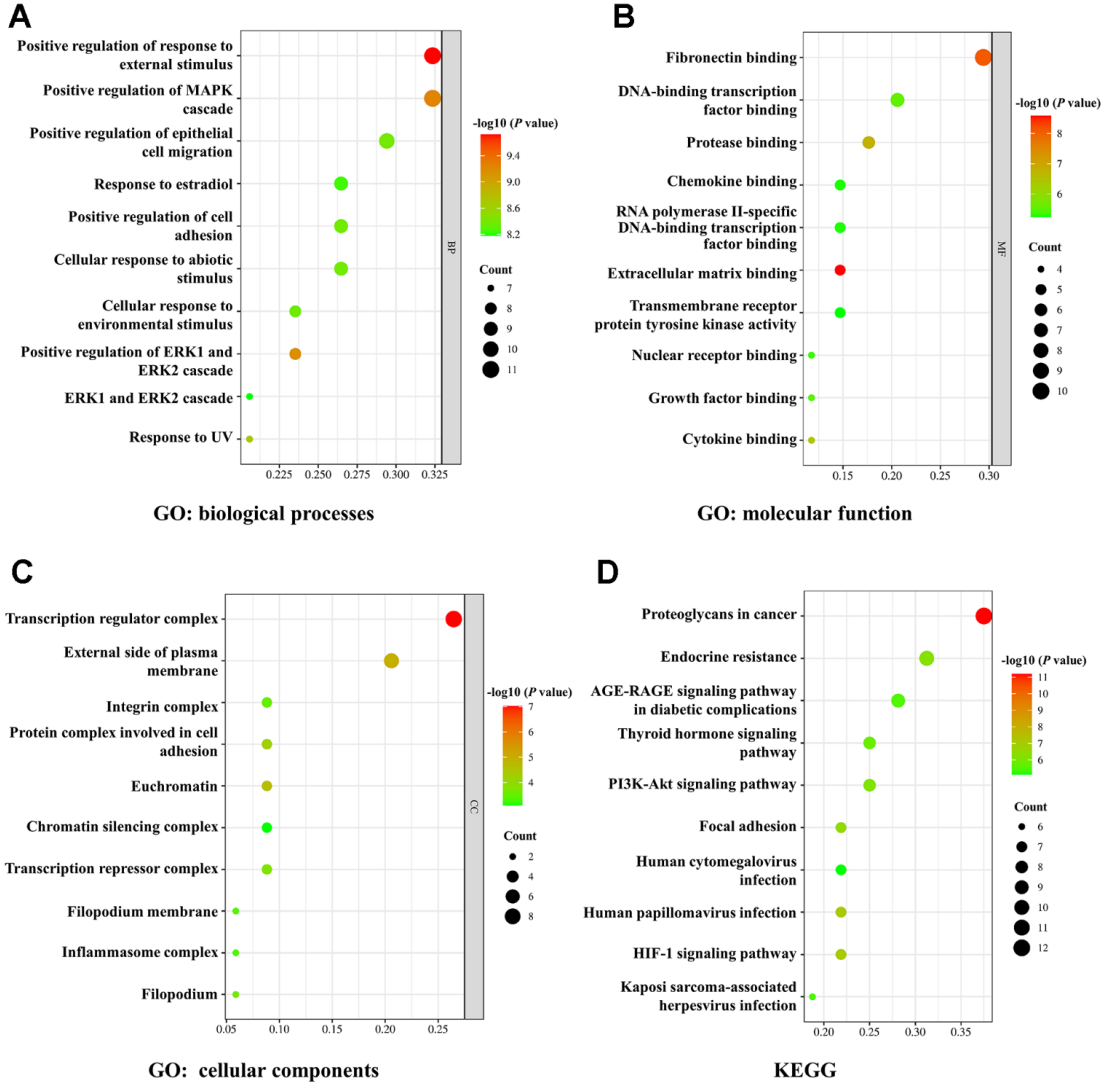
**GO and KEGG enrichment analysis.** (**A**–**C**) The bubble diagram of GO analysis for biological processes (**A**), molecular function (**B**), and cellular components (**C**). (**D**) The bubble diagram of KEGG pathway analysis. The node size indicates the gene number. The node color represents the *P*-value.

To further demonstrate the interaction of the 17 potential targets, the STRING website was used to analyze the potential targets (Figure 3A). Then the PPI network was constructed by the Cytoscape 3.8.2 (Figure 3B), and the top 10 hub targets in the PPI network were CCND1, ESR1, STAT3, SMAD2, VEGFA, MMP2, KDR, CCL2, ITGB1 and TIMP1.

### GO and KEGG enrichment analysis

**Figure 4:**
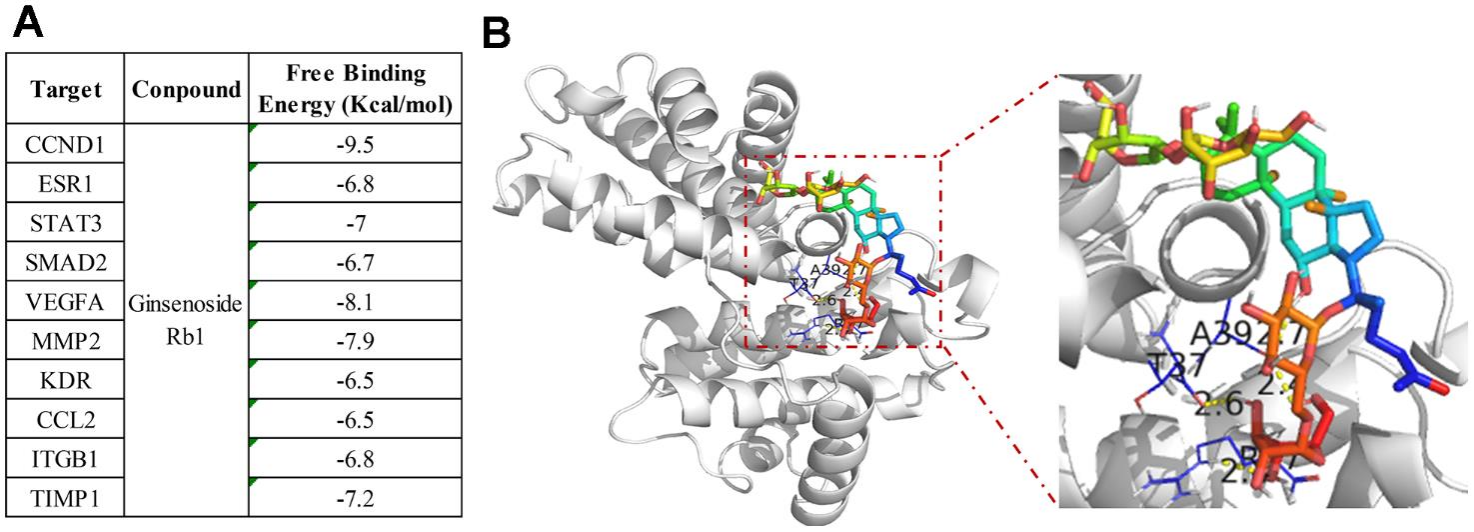
**Molecular docking between ginsenoside Rb1 and core targets protein.** (**A**) The binding energy of molecular docking. (**B**) CCND1 and ginsenoside Rb1 molecular docking visualization.

Then we used GO and KEGG enrichment analysis to further evaluate the underlying mechanisms of ginsenoside Rb1 in treating atherosclerosis. The top 10 GO enrichment terms of biological processes, molecular function, and cellular components were presented in a bubble diagram. The biological processes mainly involved positive regulation of response to external stimulus, positive regulation of MAPK cascade, response to estradiol, positive regulation of cell adhesion and cellular response to environmental stimulus (Figure 4A). The molecular function mainly involved fibronectin binding, protease binding, chemokine binding, extracellular matrix binding and cytokine binding (Figure 4B). Additionally, the cellular components were primarily associated with transcription regulator complex, integrin complex, protein complex involved in cell adhesion, transcription repressor complex and inflammasome complex (Figure 4C). KEGG pathway enrichment analysis was performed to screen potential targets and the results indicated that ginsenoside Rb1 modulates atherosclerosis development by multiple proliferation-related pathways, such as PI3K-Akt signaling pathway, thyroid hormone signaling pathway, proteoglycans in cancer, and AGE-RAGE signaling pathway in diabetic complications (Figure 4D).

### Molecular docking

**Figure 5:**
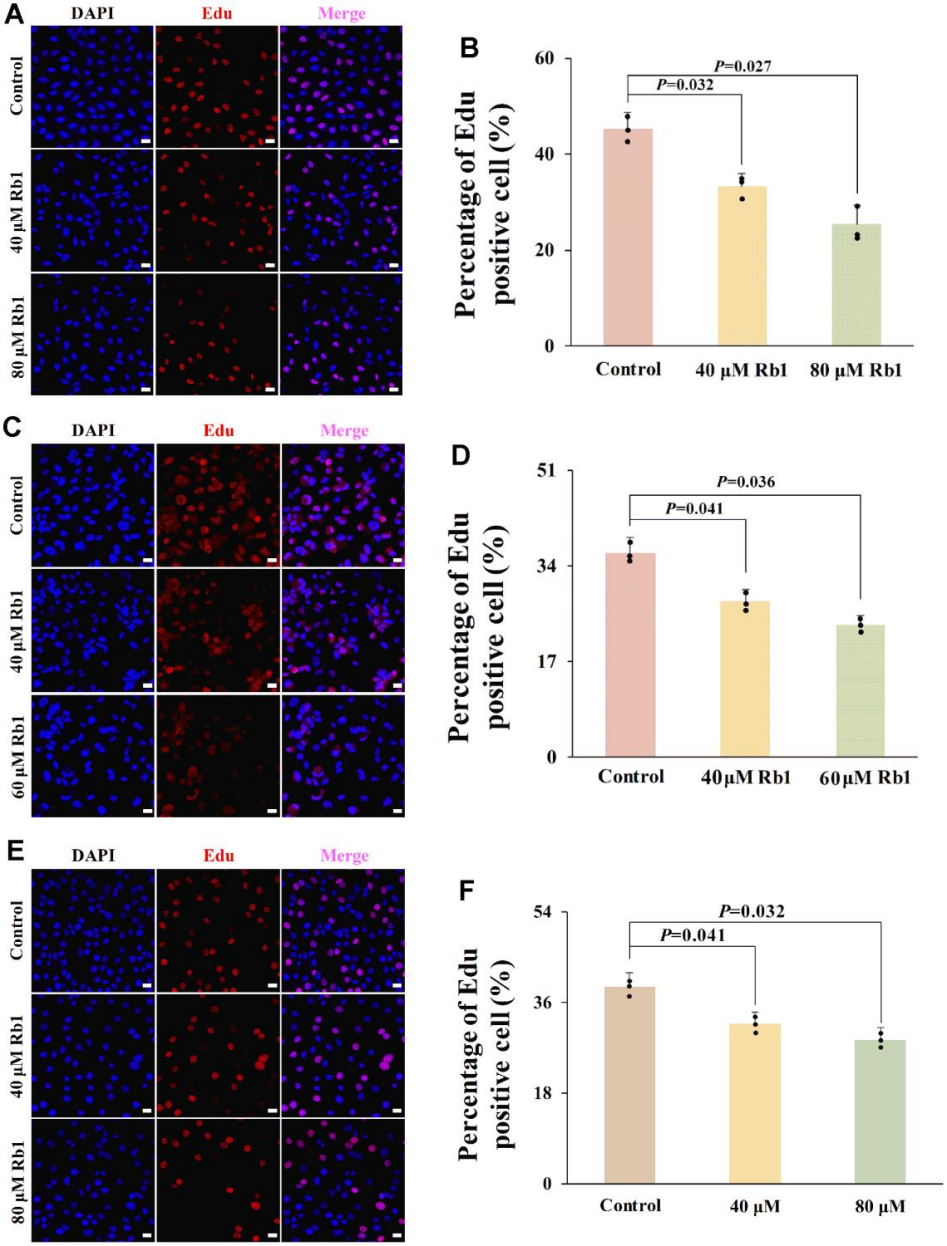
**Ginsenoside Rb1 inhibited cell proliferation of HUVECs, PBMCs and VSMCs.** (**A**) Representative Edu staining images of HUVECs. (**B**) Statistical results of Edu staining in (**A**) (n=3). (**C**) Representative Edu staining images of PBMCs. (**D**) Statistical results of Edu staining in (**C**) (n=3). (**E**) Representative Edu staining images of VSMCs. (**F**) Statistical results of Edu staining in (**E**) (n=3). *P*<0.05 represents a significant statistical difference; Scale indicates 25 μm.

To verify the accuracy of the interaction of ginsenoside Rb1 and top 10 hub targets, we used molecular docking to investigate the compound and target protein connection. The ginsenoside Rb1 was docked with the core target proteins to get the binding energy through the Autodock1.5.6 software (Figure 5A). The binding energy less than -7 kcal/mol suggests that the two has excellent binding relationship. The molecular docking results indicated that all the top 10 hub targets have particularly close binding ability with ginsenoside Rb1, and consistent with the network pharmacology results (Figure 4A). In the 10 core targets, CCND1 has the highest binding energy, suggesting that CCND1 may be the direct target of ginsenoside Rb1 in treating atherosclerosis. As shown in Figure 4B, we displayed the molecular docking pattern of CCND1 and ginsenoside Rb1.

### Ginsenoside Rb1 inhibited cell proliferation of vascular cells

CCND1 exerts a crucial role in cell proliferation by promoting the G1–S phase transition [13]. To confirm the role of ginsenoside Rb1 in vascular cell proliferation, we evaluated the ginsenoside Rb1 function in three important vascular cells proliferation, including endothelial cells, macrophages, and VSMCs. CCK-8 result showed that ginsenoside Rb1 had no obvious toxicity to HUVECs with the concentration lower than 80 μM (Supplementary Figure 1A). The Edu result indicated that 40 μM and 80 μM ginsenoside Rb1 inhibited the cell proliferation of HUVECs (Figure 5A, 5B). In PBMCs, ginsenoside Rb1 had no obvious toxicity with the concentration lower than 60 μM (Supplementary Figure 1B), and 40 μM and 60 μM ginsenoside Rb1 inhibited the cell proliferation of PBMCs (Figure 5C, 5D). In VSMCs, no obvious toxicity was observed even at concentrations of ginsenoside Rb1 up to 320 μM (Supplementary Figure 1C), but 40 μM and 80 μM ginsenoside Rb1 also showed inhibitory effects on cell proliferation (Figure 5E, 5F). The results suggested that ginsenoside Rb1 suppressed cell proliferation of vascular cells.

### Ginsenoside Rb1 inhibited the interaction of CCND1 and CDK4 complex

**Figure 6:**
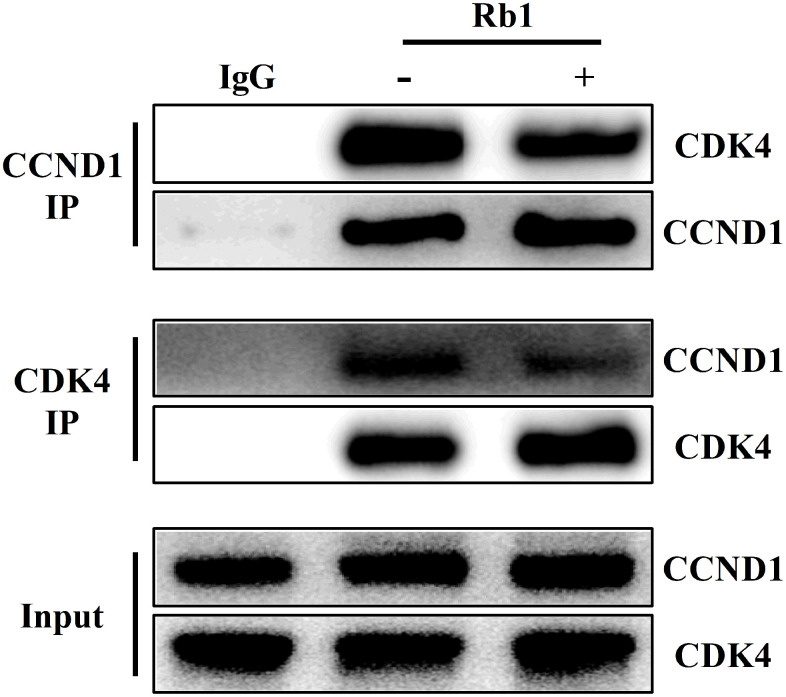
Ginsenoside Rb1 inhibited the interaction of CCND1 and CDK4 complex.

Given CCND1 promoted cell proliferation by acting synergistically with CDK4 to induce G1 to S phase transition [14], we observed whether ginsenoside Rb1 affected the interaction of CCND1 and CDK4 complex by reciprocal IP and WB analysis. WB analysis from HUVECs protein immunoprecipitated with CCND1 antibody or CDK4 antibody indicated that ginsenoside Rb1 inhibited the interaction of CCND1 and CDK4 (Figure 6).

## DISCUSSION

Atherosclerosis is the underlying cause of most CVD, which contribute to a large percentage of global morbidity and mortality [15]. Ginsenosides are mainly obtained from the plant ginseng natural steroid glycosides and triterpene saponin. Ginsenoside Rb1 is most abundant bioactive constituents in ginseng, which has properties of anti-inflammatory and anti-oxidant. Several recent studies have shown the anti-atherosclerosis effects of ginsenoside Rb1 [8–10], but its direct target is still unclear. In this study, we explored the novel anti-atherosclerosis mechanism of ginsenoside Rb1 by network pharmacology and *in vitro* experiments. Our results showed that ginsenoside Rb1 significantly inhibited the proliferation of vascular cells by inhibiting the interaction of CCDN1 and CDK4, which will provide useful insights for developing novel anti-atherosclerosis drugs by inhibiting cell proliferation.

In recent years, systems pharmacology-based strategy has become an important approach to investigating the relationship between drugs and diseases [16]. We utilized public databases to assess the overlapping targets between ginsenoside Rb1 and atherosclerosis. PPI represents the physical interactions among proteins in a cell, and these interactions are critical for all cellular processes, including signal transduction, metabolic regulation, and gene expression [17]. Hub genes with higher interaction scores were mined by PPI network and the key target CCND1 ranks higher in the PPI network, which was closely related to cell proliferation. The proliferation of three most important vascular cells, including endothelial cells, macrophages, and VSMCs is thought to play critical roles in atherosclerosis pathogenesis. The proliferation of vascular endothelial cells is closely related to neovascularization. Neovascularization in plaque is a hallmark of atherosclerotic neointimal formation and restenotic arteries [18]. Vascular endothelial growth factor induced neovascularization was meditated by promoting endothelial cell proliferation [19]. Macrophage proliferation is identified as a therapeutic target for cardiovascular disease. Clinton et al. [20] reported that replenishment of macrophages in murine atherosclerotic lesions depends predominantly on lesion macrophage proliferation rather than monocyte influx. In AopE^-/-^ mice, loss of one copy of Zinc finger protein 148 markedly caused proliferation arrest of local macrophages, which reduced atherosclerosis development [21]. In addition, VSMCs proliferation also contributes to the advanced atherosclerotic lesions development [22]. In human atherosclerosis plaques, the heat shock protein 90 is up-regulated, and heat shock protein 90 inhibition attenuates formation of atherosclerotic plaques in high fat diet-induced LDLR^-/-^ mice via suppressing VSMCs proliferation and migration [23]. Zhang et al. [24] reported that lncRNA RP4-639F20.1 was down-regulated in atherosclerotic plaques, lncRNA RP4-639F20.1 overexpression prevents the atherosclerosis development in ApoE^-/-^ mice by inhibiting the migration and proliferation of VSMCs. In this study, our results showed that ginsenoside Rb1 simultaneously inhibited the proliferation of HUVECs, macrophages and VSMCs *in vitro*. To our knowledge, there is no published paper reported that a bioactive substance could inhibit the proliferation of three kinds of vascular cells at the same time, suggesting that inhibiting cell proliferation may be the key mechanism of ginsenoside Rb1 during atherosclerosis treatment.

To validate the network pharmacology results and better understand protein-ligand interactions, we applied molecular docking simulation. Molecular docking is a structure-based method that enables the identification of novel compounds of therapeutic targets by predicting ligand–target interactions at a molecular level. The more negative the binding energy, the more stable the binding of the compound to the target [25]. Our molecular docking results showed that ginsenoside Rb1 had good binding interactions with CCND1, suggesting that ginsenoside Rb1 stably combined with CCND1 for attenuating atherosclerosis. CCND1 belongs to the cyclin family, which is highly conserved and regulates cell proliferation by promoting the G1 to S transition [26]. Mitogenic signals induce the CCND1 expression to form the CCND1-CDK4 complex [14]. The CCND1-CDK4 complex moves to the nucleus, induces the genes expression for the progression through the G1 phase [14]. In human hematopoietic stem cells, elevating the levels of CCND1–CDK4 complexes shortened the G1 cell cycle phase and promoted the G1–S phase transition [27]. Interferon-inducible transmembrane (IFITM) was up-regulated in oral squamous cell carcinoma (OSCC), and IFITM3 knockdown inhibited OSCC cell proliferation by down-regulated the complex level of CCND1-CDK4 [28]. CCND1-CDK4 overexpression prevents G1 lengthening and increases the generation and expansion of cortical progenitors, while interfering CCND1-CDK4 displays the opposite effects [29]. In mantle cell lymphoma (MCL) cells, CCND1 expression was significantly up-regulated, which accelerated the assembly of an active CCND1–CDK4 complex that drives cell cycle progression [30]. In breast cancer, lncRNA taurine-upregulated gene 1 (TUG1) was down-regulated. Overexpression of TUG1 significantly inhibited breast cancer cell proliferation by leading to cell cycle arrest, while TUG1 knockdown promoted cell cycle progression by increasing the CCND1-CDK4 complexes expression [31]. In this study, the IP results showed that ginsenoside Rb1 inhibited the interaction of CCDN1 and CDK4, which clarified the mechanism of Rb1 in anti-proliferation and further confirmed the credibility of molecular docking result.

## CONCLUSIONS

In conclusion, the network pharmacology identified 17 potential therapeutic targets of ginsenoside Rb1 in treating atherosclerosis and CCND1 has the highest binding energy with ginsenoside Rb1. The experimental verification demonstrated that ginsenoside Rb1 suppressed the vascular cell proliferation through inhibiting the interaction of CCDN1 and CDK4. Our study also provides evidence that the therapeutic effect of ginsenoside Rb1 on atherosclerosis may be partly achieved by inhibiting vascular cell proliferation.

## MATERIALS AND METHODS

### Bioinformatics analysis identify putative targets

Potential targets of ginsenoside Rb1 identification were conducted by Super-PRED (https://prediction.charite.de/subpages/target_prediction.php, accessed on January 8, 2023) and Swiss Target Prediction (http://swisstargetprediction.ch/, accessed on January 8, 2023). The transformation of the target protein names to the corresponding gene symbols was conducted by the UniProt database (https://www.uniprot.org/, accessed on January 9, 2023). “Atherosclerosis” was used as the keyword to search for related targets of this disease in the DigSee database (https://digsee.com/, accessed on January 10, 2023), GeneCards database (https://www.genecards.org/, accessed on January 10, 2023) and DisGeNET database (https://www.disgenet.org/home/, accessed on January 10, 2023). Combined the target in the three databases and then deleted the duplicated items to obtain the final atherosclerosis targets. Downloaded differentially expressed genes (DEGs) of human atherosclerotic plaque from the GEO database (http://www.ncbi.nlm.nih.gov/geo/, series: GSE202625, accessed on January 10, 2023). The differential genes between the disease group and the control group were screened by limma package, adjusting for | log 2 (fold change) | > 1 and *p* < 0.05. Venn diagram was used to take intersection of disease-related genes, active component targets, and differential genes selected above.

### Protein-protein interaction (PPI) network analysis

The potential targets were imported to the STRING database (https://string-db.org/, accessed on March 8, 2023), the species was set to be “Homo Sapiens”, and the combined score > 0.7. Imported the screened data to Cytoscape 3.9, and the CytoNCA plug-in was used to perform network topology analysis. The core genes of atherosclerosis in ginsenoside Rb1 treatment were subsequently obtained.

### Gene ontology (GO) and Kyoto Encyclopedia of Genes and Genomes (KEGG) analysis

The DAVID 6.8 database (https://david.ncifcrf.gov/, accessed on March 10, 2023) was used to perform to screen intersection targets by GO and KEGG analysis. The filtering criteria were set as “Homo Sapiens”. The GO functional analysis was performed for three categories, including the cellular component, molecular function, and biological process. The online tool of “bioinformatics” was used to visualize GO and KEGG enrichment results as a bubble chart. The false discovery rate < 0.05 represents significance.

### Cell culture

Human umbilical vein endothelial cells (HUVECs) were bought from ATCC (Manassas, VA, USA) and cultured in Endothelial Cell Growth Medium (Gibco, Waltham, MA, USA). Human peripheral blood mononuclear cells (PBMCs) were isolated from health participants according to the manufacturer’s protocol of Human Peripheral Blood Monocytes Separation Kit (Solarbio, Beijing, China). In brief, 50 mL fresh peripheral blood was mixed with 50 mL phosphate-buffered saline (PBS), and then gently layered over 50 mL Ficoll (Gibco, USA). Samples were centrifuged at 400g for 30 min without brake. After centrifugation, PBMCs in the white layer were carefully transferred to a new tube. After being washed with PBS twice, samples were then centrifuged at 400g for 10 min, and the pellets were resuspended with culture medium and counted by flow cytometry (Merck Millipore, Darmstadt, Germany). Human vascular smooth muscle cells (VSMCs) were bought from ATCC, and cells were cultured in DMEM culture medium (Gibco, USA). In addition, 10% (v/v) heat-inactivated FBS, 100 U/mL penicillin and 100 μg/mL streptomycin were added to this medium. All three cell lines used were cultured in a humidified incubator at 37° C and 5% CO_2_. In all experiments, the incubation media of vehicle- and substance-treated cells contained the same amount of solvent.

### CCK8 assays

The cell viability of different concentration ginsenoside Rb1 treated groups was assessed by a CCK-8 kit (Jiancheng, Nanjing, China). A total of 6 × 10^3^ cells were seeded in 96-well plates with corresponding treatment. After 24 hours of incubation at 37° C, 5% CO_2_, 10 μL CCK8 was added to each well and the cells were cultured for another 2 hours at 37° C, 5% CO_2_. The OD value was measured at a wavelength of 450 nm.

### Edu assays

Cell proliferation capacity was measured by an Edu Kit (RiboBio, Guangzhou, China). Cells were seeded in 12-well plates at 2 × 10^5^ cells per well. After incubation with different concentration ginsenoside Rb1 at 37° C for 24 hours, the cells were incubated with 100 mM of Edu reagent at 37° C for another 2 hours. After fixing the cells, we photographed them by the fluorescence microscope (Nikon, Tokyo, Japan).

### Co-immunoprecipitation

The HUVECs were cultured in 10 cm dish. After incubation with 40 μM ginsenoside Rb1 at 37° C for 24 hours, cells were lysed and collected protein was used for IP. The co-immunoprecipitation was conducted by commercial kit according to the manufacturer’s instruction (Thermo Fisher Scientific, Waltham, MA, USA). According to the manufacturer’s instructions, prewash beads two times with 1X Modified Coupling Buffer and bind with 5 μg cyclin D1 rabbit mAb (bsm-52046R, dilution ratio 1:200, Bioss, USA) or CDK4 mouse mAb (bsm-52028M, dilution ratio 1:200, Bioss, USA) specific primary antibody for 15 min. Next, wash beads three times with 1X Modified Coupling Buffer and crosslink antibody to beads with DSS for 30 min. Then, wash beads three times with Elution Buffer followed by two washes with IP Lysis/Wash Buffer. Incubate 7 mg cell protein with antibody-crosslinked beads overnight at 4° C. At last, wash beads two times with IP Lysis/Wash Buffer and one time with ultrapure water, elute bound antigen. The recovered proteins were measured by Western blotting (WB).

### Statistical analysis

All data are presented as the mean ± standard error of the mean (SEM) of three independent experiments. The unpaired Student’s t-test was used for *p*-value calculations by SPSS 22 (IBM Knowledge Center, Chicago, IL, USA).

### Availability of data and materials

The data sets generated during and/or analyzed during the current study are available from the corresponding author upon reasonable request.

## Supplementary Material

Supplementary Figure 1
