# Supplementary material for: Exploring the anti-atherosclerosis mechanism of ginsenoside Rb1 by integrating network pharmacology and experimental verification
Source: Aging (Albany NY). 2024 Mar 27;16(8):6745–56. doi: 10.18632/aging.205680 (PMC11087090; doi:10.18632/aging.205680)
Supplement: Supplementary Figure 1 [file aging-16-205680-s001.pdf]

SUPPLEMENTARY FIGURE

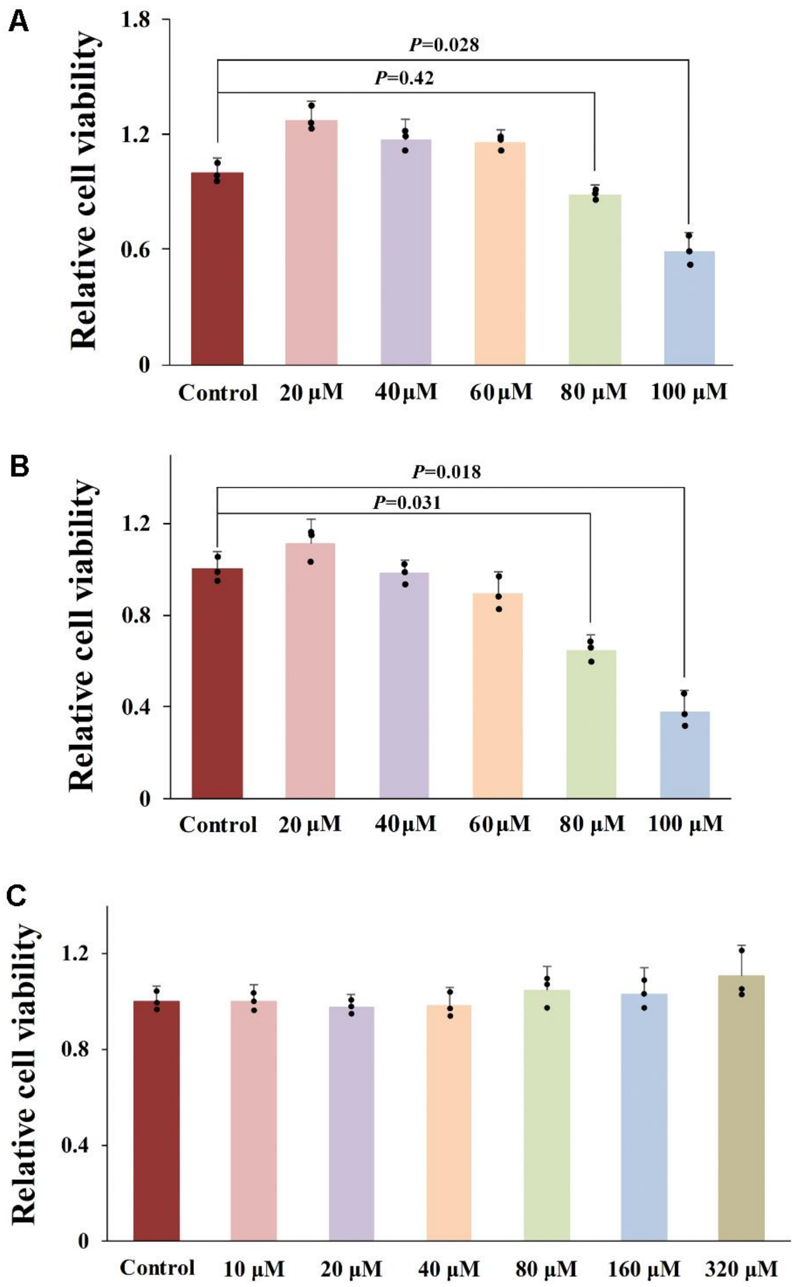

Supplementary Figure 1. The ginsenoside Rb1 effect on cell viability was detected by CCK-8. (**A**) Cell viability of endothelial cells treated with ginsenoside Rb1. (**B**) Cell viability of macrophages treated with ginsenoside Rb1. (**C**) Cell viability of vascular smooth muscle cells treated with ginsenoside Rb1.
